# Durability of immune responses to mRNA booster vaccination against COVID-19

**DOI:** 10.1172/JCI167955

**Published:** 2023-05-15

**Authors:** Prabhu S. Arunachalam, Lilin Lai, Hady Samaha, Yupeng Feng, Mengyun Hu, Harold Sai-yin Hui, Bushra Wali, Madison Ellis, Meredith E. Davis-Gardner, Christopher Huerta, Kareem Bechnak, Sarah Bechnak, Matthew Lee, Matthew B. Litvack, Cecilia Losada, Alba Grifoni, Alessandro Sette, Veronika I. Zarnitsyna, Nadine Rouphael, Mehul S. Suthar, Bali Pulendran

**Affiliations:** 1 Institute for Immunity, Transplantation, and Infection, Stanford University School of Medicine, Stanford University, Stanford, California, USA.; 2 Department of Pediatrics, Emory Vaccine Center, Emory National Primate Research Center, Atlanta, Georgia, USA.; 3 Hope Clinic of the Emory Vaccine Center, Department of Medicine, Division of Infectious Diseases, Emory University School of Medicine, Decatur, Georgia, USA.; 4 Center for Infectious Disease and Vaccine Research, La Jolla Institute for Immunology, La Jolla, California, USA.; 5 Department of Medicine, Division of Infectious Diseases and Global Public Health, University of California, San Diego, La Jolla, California, USA.; 6 Department of Microbiology and Immunology, Emory University School of Medicine, Atlanta, Georgia, USA.; 7 Department of Microbiology and Immunology and; 8 Department of Pathology, Stanford University School of Medicine, Stanford University, Stanford, California, USA.

**Keywords:** Vaccines, Adaptive immunity

## Abstract

**Background:**

Maintaining durable immunity following vaccination represents a major challenge, but whether mRNA booster vaccination improves durability is unknown.

**Methods:**

We measured antibody responses in 55 healthy adults, who received a booster dose of the Pfizer-BioNTech or Moderna vaccine against SARS-CoV-2 and calculated the half-life of the antibody titers. We also measured memory B and T cell responses in a subset of 28 participants. In 13 volunteers who received a second booster vaccine, we measured serum antibody titers and memory B and T cell responses.

**Results:**

The booster (third immunization) dose at 6 to 10 months increased the half-life of the serum–neutralizing antibody (nAb) titers to 76 days from 56 to 66 days after the primary 2-dose vaccination. A second booster dose (fourth immunization) a year after the primary vaccination further increased the half-life to 88 days. However, despite this modestly improved durability in nAb responses against the ancestral (WA.1) strain, there was a loss of neutralization capacity against the Omicron subvariants BA.2.75.2, BQ.1.1, and XBB.1.5 (48-, 71-, and 66-fold drop in titers, respectively, relative to the WA.1 strain). Although only 45% to 65% of participants demonstrated a detectable nAb titer against the newer variants after the booster (third dose), the response declined to below the detection limit in almost all individuals by 6 months. In contrast, booster vaccination induced antigen-specific memory B and T cells that persisted for at least 6 months.

**Conclusion:**

The durability of serum antibody responses improves only marginally following booster immunizations with the Pfizer-BioNTech or Moderna mRNA vaccines.

## Introduction

Recent studies have shown the declining efficacy of the Pfizer-BioNTech (BNT162b2) and Moderna (mRNA1273) mRNA vaccines after primary and booster vaccinations (1, 2). Although the booster vaccination (third dose) was effective in protecting against severe disease (3, 4), vaccine effectiveness against symptomatic disease declined rapidly to approximately 50% in the real world as a result of the emergence of the Omicron subvariants BA.4/BA.5 (1). The fourth dose showed no significant increase in efficacy against infection as compared with the third dose, suggesting that further booster immunizations may only have a marginal benefit (5). The underlying immunological basis for the declining efficacy is unknown.

We and others have demonstrated a rapid decline in serum antibody titers following a 2-dose primary vaccination with BNT162b2 (6) or mRNA1273 (7, 8), with a considerable number of individuals demonstrating weak or no neutralizing antibody (nAb) response against immune-evasive viral variants. The half-life of the serum nAb response was estimated to be 56 to 66 days up to 6 months after 2 doses of BNT162b2 or mRNA1273 (6, 8, 9). Recent data indicate that the decline in antibody response may be slower after the third dose as compared with the second dose (10); however, breakthrough infections significantly influence the antibody kinetics (11, 12). Here, we systematically evaluated the magnitude and durability of binding and nAb responses as well as memory T and B cell responses after the third and fourth doses of mRNA vaccination to determine the immunological mechanisms of declining vaccine efficacy.

## Results

### Study design and participants.

**Figure 1:**
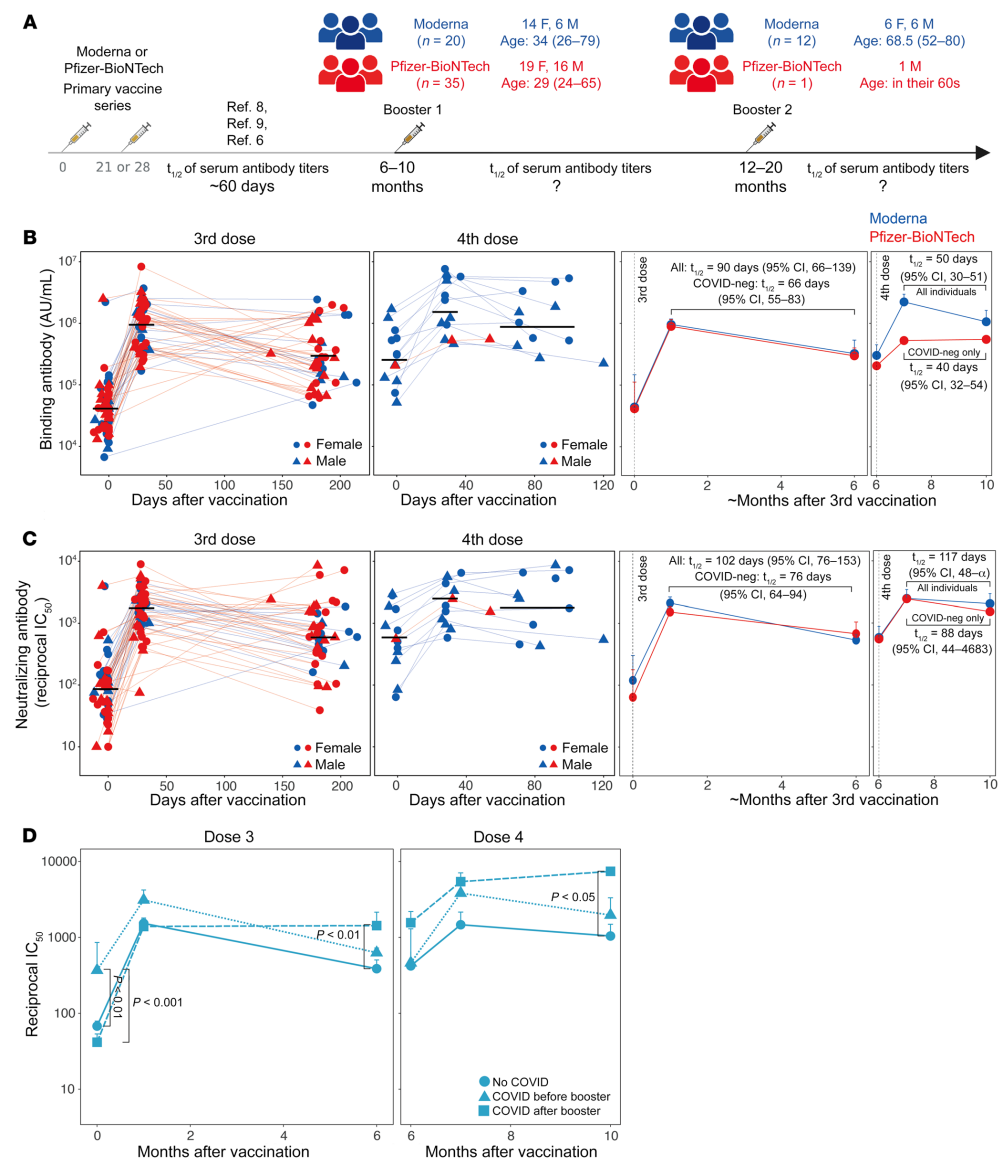
Serum antibody responses following mRNA booster vaccinations. (**A**) Schematic of the study design and participants’ details. The schematic was made using BioRender. (**B** and **C**) Anti–spike-binding IgG (**B**) and live-virus nAb titers (**C**) against the ancestral WA.1 strain. Each symbol represents an individual in the 2 plots on the left (*n* = 55 and *n* = 13 for the 3- and 4-dose groups, respectively). The black horizontal lines indicate geometric mean titers. The 2 graphs on the right show a summary (geometric mean + SEM) of the antibody responses. (**D**) nAb titers in groups of participants stratified by exposure to COVID-19. Data shown are the geometric mean for each group + SEM. The statistical difference between groups at each time point was analyzed by the Mann-Whitney *U* test. F, female; M, male; neg, negative.

We recruited 55 volunteers who received a BNT162b2 or mRNA1273 booster vaccination 6 to 10 months after completion of the primary series. Of these 55 individuals, 20 received mRNA1273 and 35 received BNT162b2. Additionally, we recruited 13 individuals who received their fourth dose 12 to 20 months after the second dose and 6 to 8 months after the third dose. Of these 13 individuals, all but 1 received mRNA1273, and all were immunocompetent. A schematic of the study design is shown in Figure 1A. The age, sex, race, vaccination, and breakthrough infections details of the participants are presented in Supplemental Table 1 (supplemental material available online with this article; https://doi.org/10.1172/JCI167955DS1). In addition to the clinical report of infections, we measured anti-nucleocapsid (anti-N) antibody responses in all individuals to determine potential undiagnosed infection with SARS-CoV-2 (Supplemental Figure 1, A and B). Thirty-one of the 55 volunteers who received the third dose and 7 of the 13 who received the fourth dose were determined to be SARS-CoV-2 naive. Of those individuals in the 3-dose cohort who had COVID-19, ten participants had an anti-N response prior to the booster, and 14 had a response after the booster. In the 4-dose cohort, 3 participants each had COVID-19 before and after the final booster.

### Durability of antibody responses to booster vaccinations.

Booster vaccination (third dose) elicited anti–spike-binding IgG titers in all individuals (Figure 1B, left panel). The geometric mean titers (GMTs) increased 21-fold from 0.43 × 10^5^ AU/mL at baseline to 9.18 × 10^5^ AU/mL in the first month. There was a 3-fold drop in the titers at 6 months when the GMT was 3 × 10^5^ AU/mL. The GMT at baseline for the individuals who received their fourth dose (approximately 6 months after their third dose; Figure 1A) was 2.9 × 10^5^ AU/mL, almost the same as that of the 6-month time point in the 3-dose group. The titers increased 6.7-fold (GMT: 19.9 × 10^5^ AU/mL) one month after the booster and decreased 2-fold (GMT: 10 × 10^5^ AU/mL) during the 3- to 4-month follow-up period (Figure 1B, middle left panel). There was no significant difference in the responses between mRNA1273 and BNT162b2 (Figure 1B, right 2 panels). The half-life of binding antibody titers estimated using the exponential decay model was 90 days when all individuals were considered (Figure 1B, middle right panel). However, the half-life estimate decreased to 66 days when only those individuals defined as SARS-CoV-2 naive were considered, a finding in line with the evidence that the durability of humoral immune responses is influenced by breakthrough infection (11). The half-life estimates after the fourth dose were 50 days or 40 days when considering all individuals and SARS-CoV-2–naive individuals, respectively (Figure 1B, right panel).

The live-virus nAb response was detectable against the ancestral strain at the time of the third dose in the majority of individuals with a GMT IC_50_ of 81. The titers increased 21-fold (GMT: 1,713) and were sustained at a magnitude 7-fold higher than the pre-booster titers (GMT: 594) (Figure 1C, left panel). The nAb titers at the peak and 6-month time points were 4.5- and 8-fold higher, respectively, than titers at the corresponding time points after 2 doses (GMTs: 343 and 49 on days 42 and 210, respectively, after the first dose) measured in our previous studies using the same assay (6, 13). The fourth dose increased the GMT to 2,477, which persisted without considerable decay (GMT: 1,997) during the follow-up period (Figure 1C, middle left panel). The half-life calculated using the exponential decay model with all the individuals was 102 days, which was substantially higher than the estimate of 56 days up to 6 months after the primary mRNA vaccine series (6). The half-life of the nAb response decreased to 76 days, when only SARS-CoV-2–naive individuals were analyzed, which was still significantly higher than the half-life of 56 days after the second dose (*P* = 0.003, Wald test). The interval between the primary vaccination and the booster did not influence the durability. The half-lives were 66 days (95% CI: 60, 73) and 76 days (95% CI: 60, 105) for individuals with an interval of 6 to 8 months or 8 to 10 months, respectively. The half-life estimates after the fourth dose were 117 days and 88 days when considering all or SARS-CoV-2–naive individuals, respectively (Figure 1C, right panel). Consistent with these estimates, the nAb response in individuals who had a breakthrough infection after the booster vaccination persisted at a significantly higher level than was observed in SARS-CoV-2–naive individuals (Figure 1D). Neither the magnitude nor the durability of responses was significantly different between males and females (Supplemental Figure 1C). Notably, there was an inverse correlation between age and the peak versus durability fold-change, but not the peak magnitude, suggesting a relatively higher persistence in older adults (Supplemental Figure 1, D and E).

For a direct comparison of the durability of antibody responses after the second, third, and fourth doses, we calculated the fold-change between peak versus the durability time points of binding as well as nAb responses after each vaccination only in SARS-CoV-2–naive individuals. The data after 2 vaccinations for BNT162b2 were previously published in our study (6). The ratio improved marginally upon subsequent booster immunizations, consistent with our half-life estimates (Supplemental Figure 1F). Whether the antibody durability after the fourth dose continues to persist out to 6 months remains to be investigated. Taken together, these data show that subsequent booster immunizations modestly improved the half-life of antibody titers, which, in conjunction with the increase in the absolute magnitude of nAb titers, resulted in an improved durability of antibody responses.

### Antibody breadth.

**Figure 2:**
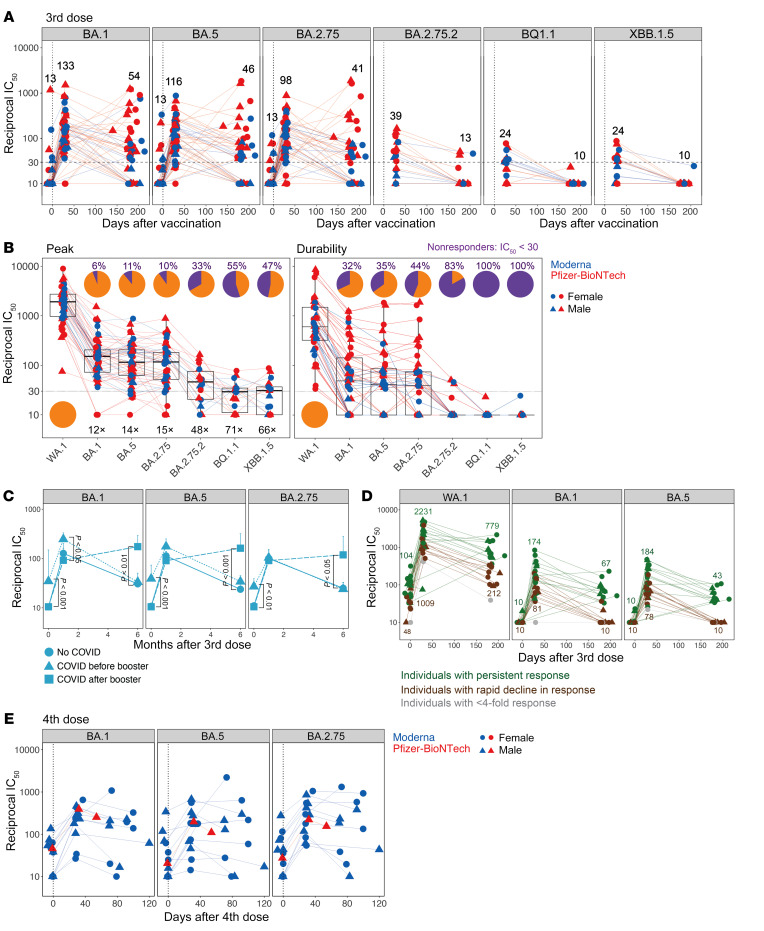
nAb breadth following booster vaccinations. (**A**) Live-virus nAb response measured against Omicron BA.1, BA.5, BA.2.75, BA.2.75.2, and BQ.1.1 variants (*n* = 55 for BA.1, BA.5, and BA.2.75; *n* = 18 for BA.2.75.2 and BQ.1.1; *n* = 17 for XBB.1.5). Participants who had the highest titers against the ancestral and Omicron BA.1 variant were selected for this assay. (**B**) nAb titers against the viruses indicated on the *x* axis at peak (left panel) and their durability (right panel). Pie charts show the proportion of participants who responded (in orange) versus those who did not (in purple) against each virus. Nonresponders were defined as those who had an IC_50_ below 30. The numbers inside the graph followed by X indicate the decrease in titers against variants in comparison with the ancestral strain. The fold change was calculated using responders, i.e., those with an IC_50_ above 30 only. Horizontal dotted lines in **A** and **B** indicate the cutoff used to define the responders. (**C**) nAb titers against the variants indicated on the plots in participants stratified by exposure to COVID-19. Data shown are the geometric mean for each group + SEM. The statistical difference between groups at each time point was analyzed by the Mann-Whitney *U* test. (**D**) nAb titers in all SARS-CoV-2–naive individuals. Each symbol represents an individual. Individuals who showed a neutralization titer below 30 against BA.1, BA.5, or BA.2.75 at 6 months were classified as those with a rapid decline (brown). Data points for individuals who showed a less than 4-fold increase in titers against the variants are shown in gray. The rest of the individuals were considered normal responders (green). (**E**) Live-virus nAb response measured against Omicron variants BA.1, BA.5, and BA.2.75 (*n* = 13) in participants who received a fourth dose of the mRNA vaccine.

We and others have reported the generation of antibodies that can neutralize Omicron following a mRNA booster vaccination (third dose) (14–17). Consistent with these studies, we observed live-virus nAb titers against Omicron BA.1, BA.5, and BA.2.75 subvariants (Figure 2A). The peak GMTs were 133, 116, and 98 against BA.1, BA.5 and BA.2.75, respectively, which was 12- to 15-fold lower than that of the ancestral strain (Figure 2A). In contrast, the GMTs against the recently emerged variants of interest — BA.2.75.2, BQ.1.1, and XBB.1.5 — which were measured in a subset of 18 individuals with the highest nAb titers against other viruses were 39, 24, and 24, respectively, with 30% to 55% of individuals having nAB titers below the limit of detection (Figure 2, A and B). These titers were approximately 5-fold lower than those against Omicron BA.1 or BA.5 and 45- to 70-fold lower than the response against the WA.1 strain (Figure 2B). By 6 months, almost no one had a detectable response against BA.2.75.2, BQ.1.1, or XBB.1.5. Notably, even against BA.1 and BA.5, approximately one-third of the vaccinees had no detectable nAb response by 6 months (Figure 2B, right panel). This observation prompted us to investigate whether a lack of exposure to SARS-CoV-2 is associated with a more rapid decline in the nAb response against variants. Our analysis showed that the nAb titers against the variants at baseline were significantly higher in individuals who had COVID-19 prior to the booster vaccination (Figure 2C). One month after the booster, all individuals had nAbs against the variants, but the response persisted durably (*P <* 0.05) in individuals who had a breakthrough infection after the booster (Figure 2C). It was notable that the nAb titers in individuals who had COVID-19 prior to the booster declined to levels statistically comparable to those of SARS-CoV-2–naive vaccinees, despite eliciting relatively higher titers following the booster. We further investigated the durability of nAbs in only SARS-CoV-2–naive participants and observed that those individuals whose nAb titers rapidly declined to below the detection limit against the variants also had lower titers against the ancestral strain at all time points (Figure 2D). Although the magnitude of nAb titers was only approximately 2-fold lower in this subgroup at the peak of the response, the decline in response was rapid, resulting in weak, and, in the case of the variants, no neutralization titers at 6 months. Finally, we also measured nAb titers against BA.1, BA.5, and BA.2.75 after the fourth dose (Figure 2E). The variant-specific nAb titers were induced more moderately by the fourth dose and persisted stably until the follow-up period at 3 to 4 months (Figure 2E).

### Cellular immune responses.

**Figure 3:**
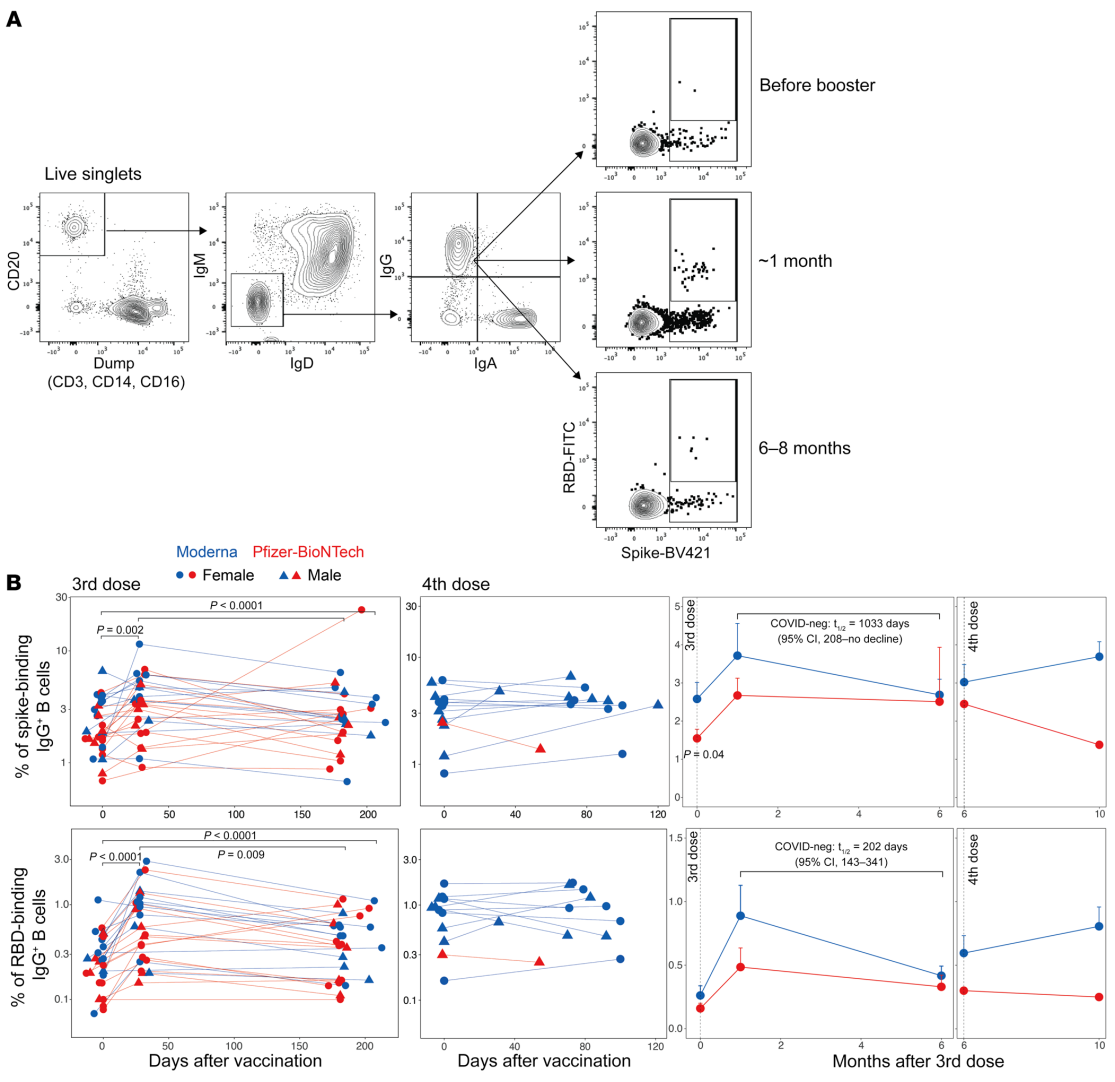
Memory B cell responses to the booster vaccination. (**A**) Representative flow cytometry profile showing the gating strategy to define spike-specific B cell frequencies (gated as live CD20^+^IgD^–^IgM^–^spike^+^ RBD^+/–^ cells. (**B**) Frequency of WA.1 spike–specific (top panel) or RBD-specific (bottom panel) memory B cells relative to CD20^+^IgD^–^IgM^–^ B cells. Each symbol represents an individual (*n* = 28, after the third dose; *n* = 13, after the fourth dose). The statistical differences between time points were determined using the Wilcoxon matched-pairs, signed-rank test. The 2 graphs on the right show a summary of the responses (geometric mean + SEM). The statistical difference between the groups was determined using the Mann-Whitney *U* test.

Next, we assessed spike-specific memory B cells by flow cytometric analysis of PBMCs labeled with ﬂuorescence-tagged recombinant spike and receptor-binding domain (RBD) proteins (Figure 3A). The third-dose vaccination significantly increased the frequency of spike-binding memory B cells from 1.9% (of CD20^+^IgM^–^IgD^–^IgA^–^IgG^+^) at baseline to 3.2% at approximately 1 month. The response persisted durably and was maintained at 2.6% six months later (Figure 3B and Supplemental Figure 2A, top left panel). The frequency of RBD^+^ memory B cells followed the same kinetics but was present at a lower magnitude compared with spike-binding B cells (Figure 3B and Supplemental Figure 2A, bottom panel). Although we did not have the appropriate peak time point to observe a potential increase in the magnitude after the fourth dose, the memory B cell frequency persisted at the prevaccination magnitude by 3 to 4 months (Figure 3B and Supplemental Figure 2A). The memory B cell frequencies were not significantly associated with sex (Supplemental Figure 2B).

**Figure 4:**
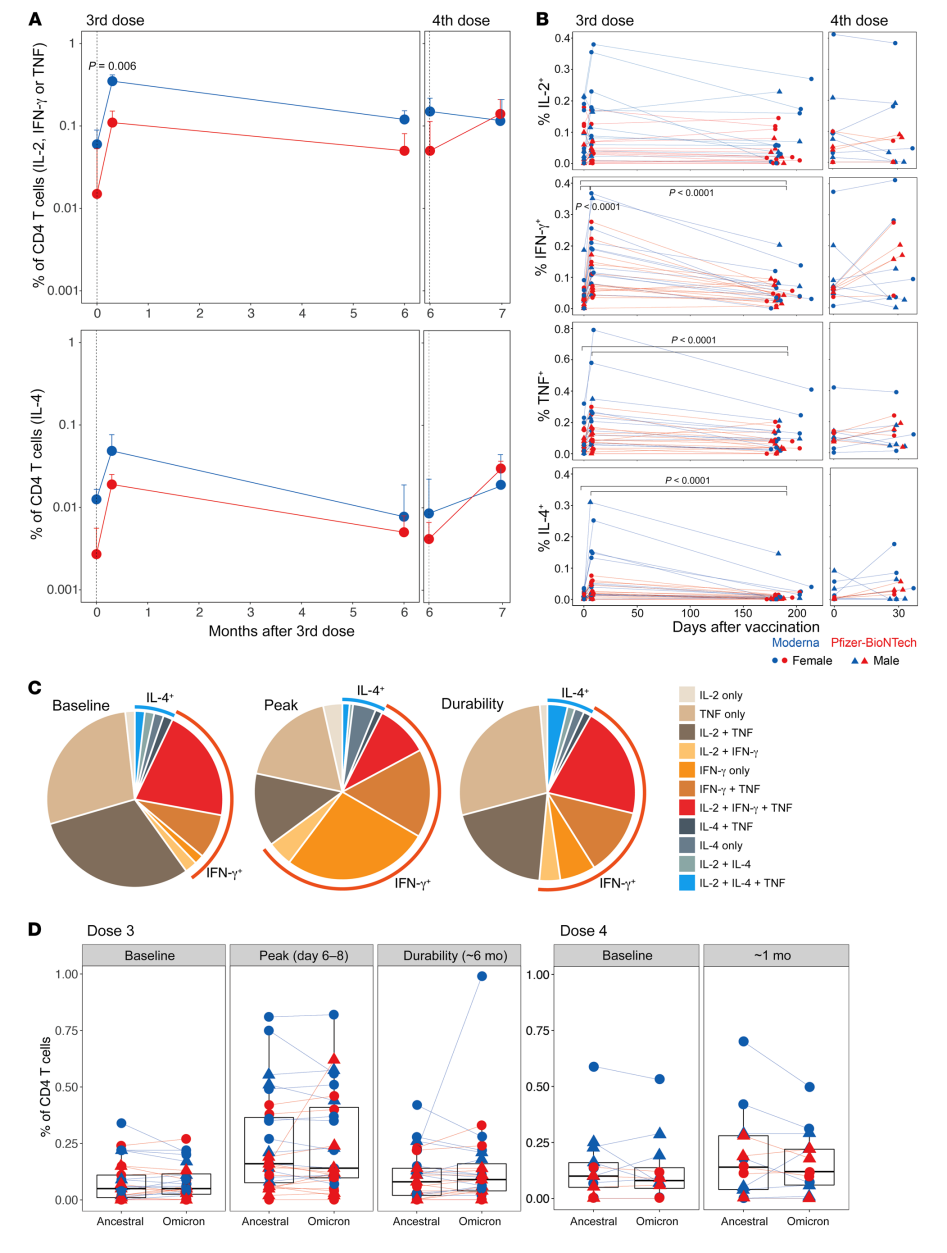
T cell responses induced by the mRNA booster vaccination. (**A**) Summary of the frequency of ancestral spike–specific CD4^+^ T cells secreting IL-2, IFN-γ, or TNF (Th1-type, top panel) and IL-4 (Th2-type, bottom panel). Median responses ± SEM are plotted. The statistical difference between groups was determined using a Mann-Whitney *U* test. (**B**) Frequency of spike-specific CD4^+^ T cells that produced the indicated individual cytokines (geometric mean + SEM). Each symbol represents an individual (*n* = 28 after the third dose and 13 after the fourth dose). The statistical significance between time points was calculated using a Wilcoxon matched-pairs, signed-rank test. (**C**) Pie charts showing the proportion of spike-specific CD4^+^ T cells producing 1, 2, or 3 cytokines in response to the third dose of the vaccine. (**D**) Comparison of CD4^+^ T cell frequencies (Th1-type producing IL-2, TNF, or IFN-γ) between ancestral and Omicron BA.1 viral strains measured at the time points indicated on the plots.

We also measured T cell responses using an intracellular cytokine staining (ICS) assay following a 6-hour stimulation of PBMCs with overlapping peptide pools spanning the spike proteins of the ancestral and Omicron variants (18). The booster vaccination induced significant CD4^+^ T cell responses, primarily Th1-type (Figure 4A), consistent with previous studies (13, 19). The magnitude of response approximately 7 days after vaccination was significantly higher with mRNA1273 vaccination than with BNT162b2 vaccination. The response at the pre-booster time point involved predominantly IL-2^+^ and TNF^+^CD4^+^ T cells with or without IFN-γ, suggesting an establishment of potent memory T cells during previous vaccinations. While the frequency of cells producing IL-2 and TNF remained elevated after vaccination, the cells producing only IFN-γ were significantly induced by day 7 (Figure 4B), suggesting a potential differentiation of memory T cells into an effector phenotype. The frequency of IFN-γ–, TNF-, and IL-4–producing CD4^+^ T cells, albeit much lower, persisted out to 6 months at a frequency higher than that observed before the booster (Figure 4B), but the proportion of cells producing multiple cytokines, which comprised predominantly IL-2^+^TNF^+^ or IL-2^+^TNF^+^IFN-γ^+^ cells, largely returned to the baseline state (Figure 4C). The frequencies of CD4^+^ T cells recognizing ancestral or Omicron spike antigens were relatively comparable, suggesting a conservation of T cell epitopes consistent with previous studies (Figure 4D) (18, 20, 21). The booster vaccination also elicited CD8^+^ T cells producing IFN-γ by day 7, and the frequency of these cells returned to pre-booster levels by 6 months (Supplemental Figure 3, A and B). The T cell responses were also not significantly different between males and females and showed an overall positive, but statistically insignificant, correlation with the durability of antibody responses (Supplemental Figure 3, C–E). Collectively, these data demonstrate that the booster vaccination reactivated memory B and T cell responses and maintained the durable memory response elicited by prior vaccinations.

## Discussion

mRNA vaccines stimulate durable immune memory (9); however, serum antibody responses steadily decline, with a half-life of 56 to 66 days in the first 6 months after 2 doses (6–8), resulting in weak to undetectable nAb titers in many individuals. Concurrent with declining antibody titers, the effectiveness of vaccine-mediated protection against infection declines in the population (22, 23). The emergence of immune-escape variants such as Omicron further reduces vaccine effectiveness, mandating booster vaccinations (24). Whether the durability of immune responses, in particular, the half-life of serum antibody titers improves after subsequent booster vaccinations, was the primary question addressed in this study.

Our data show that a booster vaccination (third dose) increased the half-life of binding and nAb titers only marginally when SARS-CoV-2–naive participants were analyzed. While the increase in *t_1/2_* of the binding antibody titer was not statistically different, the increase in *t_1/2_* of the nAb titer to 76 days from 56 days after the second vaccination in our previous study (6) was statistically significant (*P* = 0.003 between the decay rates after the second and third vaccinations, Wald test). We used seropositivity to SARS-CoV-2 nucleocapsid to exclude the potential interference of breakthrough infections in our durability calculations. However, our results should be considered with caution, given the caveat that a lack of an anti-N response does not preclude the possibility of a breakthrough infection. Whether the marginal increase in half-life is truly an increase in durability associated with a vaccine-induced increase in bone marrow plasma cells (BMPCs) warrants investigation. Evidence indicates the generation of BMPCs that were detectable 6 months after 2 doses of BNT162b2; however, the magnitude was modest (median 0.06% of total IgG-producing BMPCs) in contrast to 1.4% against seasonal influenza or 0.15% against tetanus antigen (25). While it is conceivable that BMPCs accumulate and increase in number after each subsequent booster vaccination, resulting in a slow but cumulative improvement in durability, it is unknown if the BMPCs induced by mRNA vaccination have life spans as long as those induced by live-attenuated viral vaccines such as smallpox and yellow fever vaccines.

A secondary objective of our study was to determine the nAb response against emerging SARS-CoV-2 variants. The booster vaccination induced a nAb response against the Omicron subvariants BA.1, BA.5, and BA.2.75, consistent with previous reports (15, 26, 27). However, the responses against the most recently emerged BA.2.75.2, BQ.1.1, and XBB.1.5 variants with the concerning R346T mutation were strikingly diminished. By 6 months, almost no one had a detectable titer against these variants. These findings, alongside our recent data indicating enhancement of breadth against these variants following a bivalent booster containing ancestral and BA.5 spike (28), suggest that monovalent boosters may be ineffective in protecting against infection, and thus bivalent vaccines are required for subsequent boosters to improve the serological responses. In contrast, the memory T and B cell responses were durable after the booster vaccinations, continuing to provide protection against severe disease.

The limitations of the present study include a smaller cohort size as well as a shorter follow-up period (3–4 months versus 6 months) after the fourth dose, limited by the unavailability of monovalent mRNA vaccines. It should also be noted that the individuals who received the fourth dose were relatively older than the third-dose vaccinees, although all participants were immunocompetent. We also did not evaluate the effect of the bivalent booster in this study.

In summary, our comprehensive analysis of immune responses provides evidence that a lack of nAb responses against emerging variants despite the marginal improvement of durability against the ancestral strain underlies the declining efficacy of booster doses of mRNA vaccines and will aid in decision-making regarding the utility of subsequent booster vaccinations.

## Methods

### ECL binding ELISA for anti-spike and anti-N antibodies.

Anti-spike and anti-N protein IgG titers were measured using V-plex SARS-CoV-2 panels 29 (catalog K15624U) and 2 (catalog K15383U), respectively, from Meso Scale Diagnostics (MSD). The assay was performed according to the manufacturer’s instructions. Briefly, the multispot, 96-well plates were blocked in 0.15 mL blocking solution with shaking at 700 rpm at room temperature. After 30 minutes of incubation, 50 μL serum samples were diluted in antibody diluent solution, and serially diluted calibrator solution was added to each plate in the designated wells and incubated at room temperature for 2 hours with shaking. After 2 hours of incubation, the plates were washed, and 50 μL SULFO-TAG–conjugated anti-IgG (MSD) was added, and the plates were incubated at room temperature for 1 hour. After incubation, the plates were washed, and 0.15 mL MSD GOLD Read Buffer was added. The plates were immediately read using the MSD instrument. The unknown concentrations were extrapolated using a standard curve drawn using the calibrators in each plate and presented as relative MSD AU/mL or BAU/mL.

### Viruses and cells for the focus reduction neutralization test.

VeroE6-TMPRSS2 cells were cultured in complete DMEM (DMEM with 10% FBS plus penicillin/streptomycin) in the presence of 10 mg/mL Gibco Puromycin (Thermo Fisher Scientific, A11138-03). nCoV/USA_WA1/2020 (WA/1) was propagated from an infectious SARS-CoV-2 clone as previously described (29). icSARS-CoV-2 was passaged once to generate a working stock. The BA.1 isolate has been previously described (15). Omicron subvariants were isolated from residual nasal swabs: the BA.5 isolate (EPI_ISL_13512579) was provided by Richard Webby (St. Jude Children’s Research Hospital) and the BA.2.75.2 (EPI_ISL_15146622), BQ.1.1 isolate (EPI_ISL_15196219), and BA.2.75 isolate (EPI_ISL_14393635) were provided by Benjamin Pinsky (Stanford University, Stanford, California, USA). All variants were plaque purified and propagated once in VeroE6-TMPRSS2 cells to generate working stocks. The XBB.1.5 isolate (EPI_ISL_16026423) was provided by Andrew Pekosz (Johns Hopkins University, Baltimore, Maryland, USA) and was passaged once on VeroE6-TMPRSS2 cells to generate a larger stock. Viruses were deep-sequenced and confirmed as previously described (30).

Focus reduction neutralization test (FRNT) assays were performed as previously described (15, 30, 31). Briefly, samples were diluted 3-fold in 8 serial dilutions using DMEM in duplicates with an initial dilution of 1:10 in a total volume of 60 μL. Serially diluted samples were incubated with an equal volume of SARS-CoV-2 (100–200 foci per well based on the target cell) at 37°C for 45 minutes in a round-bottomed, 96-well culture plate. The antibody-virus mixture was then added to VeroE6-TMPRSS2 cells and incubated at 37°C for 1 hour. After incubation, the antibody-virus mixture was removed, and 100 μL prewarmed 0.85% methylcellulose (MilliporeSigma, M0512-250G) overlay was added to each well. Plates were incubated at 37ºC for either 18 or 40 hours, and the methylcellulose overlay was removed and washed 6 times with PBS. Cells were fixed with 2% paraformaldehyde in PBS for 30 minutes. Following fixation, plates were washed twice with PBS, and permeabilization buffer (0.1% BSA and 0.1% saponin in PBS) was added to permeabilized cells for at least 20 minutes. Cells were incubated with an anti–SARS-CoV spike protein primary antibody directly conjugated to Alexa Fluor 647 (CR3022-AF647, Cell Signaling Technology) for 4 hours at room temperature or overnight at 4°C. Cells were washed 3 times in PBS, and foci were visualized on an ELISPOT reader. Antibody neutralization was quantified by counting the number of foci for each sample using the Viridot program (32). The neutralization titers were calculated as follows: 1 – (ratio of the mean number of foci in the presence of serum and foci at the highest dilution of the respective serum sample). Each specimen was tested in duplicate. The FRNT_50_ titers were interpolated using a 4-parameter nonlinear regression in GraphPad Prism, version 9.2.0 (GraphPad Software). Samples that did not neutralize at the limit of detection of 50% were plotted at 10 and used for the geometric mean and fold-change calculations.

### Antibody half-life calculations.

Mixed-effects models implemented in Monolix Suite 2021R1 (Lixoft) were used to estimate the corresponding half-lives of antigen-specific antibodies. The exponential decay model dAb/dt = –*k* × Ab was fitted to the longitudinal data starting from day 21 after the third or fourth vaccine doses, where Ab is the antibody concentration and *k* is the exponential decay. The corresponding half-lives were calculated as *t_1/2_* = ln(2)/*k* (ln denotes natural log). The individual-level parameters were lognormally distributed for the initial antibody concentration (at day 21) and normally distributed for the decay rate *k*, with an assumption of no correlations between the random effects. We assumed a multiplicative independent lognormal observation error. Estimation of the population parameters was performed using the stochastic approximation expectation maximization (SAEM) algorithm.

### Spike protein–specific memory B cell staining.

Cryopreserved PBMCs were thawed and washed twice with 10 mL FACS buffer (1x PBS containing 2% FBS and 1 mM EDTA) and resuspended in 100 μL PBS containing Zombie UV live/dead dye at a 1:200 dilution (BioLegend, 423108) and incubated at room temperature for 15 minutes. Following the washing, cells were incubated with an antibody cocktail for 1 hour on ice, protected from light. The following antibodies were used: IgD phycoerythrin (PE) (Southern Biotech, 2030-09), IgM peridinin chlorophyll protein-Cy5.5 (PerCP-Cy5.5) (BioLegend, 314512), CD20 allophycocyanin-H7 (APC-H7) (BD Biosciences, 560734), CD27 PE-Cy7 (BioLegend, 302838), CD14 brilliant violet 650 (BV650) (BioLegend, 301836), CD16 BV650 (BioLegend, 302042), IgG brilliant ultraviolet 496 (BUV496) (BD Biosciences, 741172), CD3 BV650 (BD Biosciences, 563916), and CD21 PE-CF594 (BD Biosciences, 563474). In addition, Alexa Fluor 647–labeled Omicron BA.1 spike (SinoBiological, 40589-V08H26), BV605-labeled Omicron BA.1 RBD (SinoBiological), BV421-labeled ancestral spike (SinoBiological, 40589-V27B-B), and FITC-labeled ancestral RBD (SinoBiological) proteins were used as probes for memory B cell staining. All antibodies were used as per the manufacturer’s instructions, and the final concentration of each probe was 0.1 μg/mL. Cells were washed twice in FACS buffer and immediately acquired on a BD FACSAria III. Flowjo software, version 10 (TreeStar), was used for data analysis.

### Intracellular cytokine staining assay.

Antigen-specific T cell responses were measured using the intracellular cytokine staining assay. Live-frozen PBMCs were revived, counted, and resuspended at a density of 2 × 10^6^ live cells/mL in complete RPMI-1640 (supplemented with 10% FBS and penicillin/streptomycin) and rested for 6 hours at 37°C in a CO_2_ incubator. After the incubation, the cells were washed once and resuspended at a density of 12 × 10^6^/mL to 15 × 10^6^/mL in complete RPMI-1640, and 100 μL cell suspension containing 1.2 × 10^6^ to 1.5 × 10^6^ cells was added to each well of a 96-well, round-bottomed tissue culture plate. Each sample was treated with 2 or 3 conditions depending on cell numbers: no stimulation or a peptide pool spanning the spike protein of the ancestral WA.1 strain or the Omicron BA.1 variant (where cell numbers permitted) in the presence of 1 μg/mL anti-CD28 (clone CD28.2, BD Biosciences) and anti-CD49d (clone 9F10, BD Biosciences) as well as anti-CXCR3 and anti-CXCR5. The details of peptide synthesis and purity have been described previously (18). Briefly, the peptide pools were 15 mer peptides with 10 mer overlaps spanning the entire spike protein sequence of each variant. The amino acids in the variant peptide pools that vary from the ancestral spike protein sequence are provided in Supplemental Table 3 in Tarke et al. (18). Each peptide was dissolved at a concentration of 20 mg/mL in DMSO, and individual peptides were pooled to prepare each variant-specific peptide pool following sequential lyophilization, as previously reported (18). Each peptide pool contained 253 peptides and was resuspended in DMSO at a concentration of 1 mg/mL. PBMCs were stimulated at a final concentration of 1 μg/mL of each peptide in the final reaction with an equimolar amount of DMSO (0.5% v/v in 0.2 mL total reaction volume) as a negative control. The samples were incubated at 37°C in CO_2_ incubators for 2 hours before addition of 10 μg/mL brefeldin A. Cells were incubated for an additional 4 hours and then washed with PBS and stained with Zombie UV fixable viability dye (BioLegend). Cells were washed with PBS containing 5% FBS, before the addition of a surface antibody cocktail. Cells were stained for 20 minutes at 4°C in a 100 μL volume. Subsequently, the cells were washed, fixed, and permeabilized with Cytofix/Cytoperm buffer (BD Biosciences, catalog 555028) for 20 minutes. The permeabilized cells were stained with intracellular cytokine–staining antibodies for 20 minutes at room temperature in 1′ perm/wash buffer (BD Biosciences, catalog 555028). Details of the antibody panel used in the assay were described previously (33). Cells were then washed twice with perm/wash buffer and once with staining buffer before acquisition using the BD Symphony Flow Cytometer and the associated BD FACSDiva software. All flow cytometric data were analyzed using Flowjo software, version 10 (TreeStar).

### Statistics.

The difference between any 2 groups at a time point was measured using a 2-tailed, nonparametric Mann-Whitney unpaired rank-sum test. The difference between time points within a group was measured using a Wilcoxon matched-pairs, signed-rank test. The correlations were Spearman’s correlations based on ranks. All statistical analyses were performed using GraphPad Prism, version 9.0.0, or R, version 3.6.1. A *P* value of less than 0.05 was used as the significance cutoff. Exponential decay rates were compared for binding and nAbs after the second and third vaccine doses using the Wald test implemented in R. For binding antibodies, the exponential decay rates estimated using mixed-effects models after the second dose (6) and after the third dose with breakthrough cases removed were 0.0124 (standard error [SE] = 0.000644) per day and 0.00913 (SE = 0.000892) per day, respectively. For nAb titers, the exponential decay rates after the second dose (6) and after the third dose with breakthrough cases removed were 0.0124 (SE = 0.000644) per day and 0.00913 (SE = 0.000892) per day, respectively. All figures were made in GraphPad Prism or R and organized in Adobe Illustrator.

### Study approval.

All participants were recruited as part of an observational study at the Hope Clinic of the Emory Vaccine Center. The participants received the COVID-19 vaccine as the standard of care. Blood samples were collected from volunteers under informed consent. The participants’ details were deidentified and are presented in Supplemental Table 1. The study was reviewed and approved by the IRB of Emory University School of Medicine (study no. 00002061).

## Author contributions

BP conceived the study. PSA and BP designed the study and were responsible for the overall conduct of the study. HS, CH, KB, SB, ML, MBL, CL, and NR collected clinical samples. PSA and YF performed binding antibody assays. LL, BW, MEDG, and ME performed neutralization assays. PSA, YF, MH, and HSH performed memory T and B cell assays. VIZ calculated antibody half-lives. AG and AS provided peptide pools for T cell assays. NR, MSS, and BP supervised the experiments. PSA formally analyzed all the data sets and prepared the figures. PSA and BP wrote the manuscript with suggestions and assistance from all co-authors. All the authors read and accepted the final contents of the manuscript.

## Supplementary Material

Supplemental data

ICMJE disclosure forms

## Version 1 03/23/2023

In-Press Preview

## Version 2 05/15/2023

Electronic publication
