# Supplementary material for: Durability of immune responses to mRNA booster vaccination against COVID-19
Source: J Clin Invest. 2023 May 15;133(10):e167955. doi: 10.1172/JCI167955 (PMC10178835; doi:10.1172/JCI167955)
Supplement: Supplemental data [file jci-133-167955-s144.pdf]

Supplemental information

Durability of immune responses to the booster mRNA vaccination against COVID-19

Prabhu S. Arunachalam,[1] Lilin Lai,[2] Hady Samaha,[3] Yupeng Feng,[1] Mengyun Hu,[1] Harold Sai-yin Hui,[1] Bushra Wali,[2] Madison Ellis,[2] Meredith Davis-Gardner,[2] Christopher Huerta,[3] Kareem Bechnack,[3] Sarah Bechnack,[3] Matthew Lee,[3] Matthew Litvack,[3] Cecilia Losada,[3] Alba Grifoni,[4] Alessandro Sette,[4,5] Veronika I. Zarnitsyna,[6] Nadine Rouphael,[3] Mehul S. Suthar,[2] Bali Pulendran[1,7,8,*]

[1]Institute for Immunity, Transplantation and Infection, Stanford University School of Medicine, Stanford University, Stanford, CA, USA.

[2]Department of Pediatrics, Emory Vaccine Center, Emory National Primate Research Center, Atlanta, GA, USA.

[3]Hope Clinic of the Emory Vaccine Center, Department of Medicine, Division of Infectious Diseases, Emory University School of Medicine, Decatur, GA 30030, USA.

[4]Center for Infectious Disease and Vaccine Research, La Jolla Institute for Immunology, La Jolla, CA 92037, USA.

[5]Department of Medicine, Division of Infectious Diseases and Global Public Health, University of California, San Diego, La Jolla, CA 92037, USA.

[6]Department of Microbiology and Immunology, Emory University School of Medicine, Atlanta, GA 30329, USA.

[7]Department of Microbiology and Immunology, Stanford University School of Medicine, Stanford University, Stanford, CA, USA.

[8]Department of Pathology, Stanford University School of Medicine, Stanford University, Stanford, CA, USA.

Lilin Lai and Hady Samaha contributed equally.

*Correspondence to Bali Pulendran: bpulend@stanford.edu.

Supplemental Table 1. Demographic information of the participants.

Participant	Age range	Gender	Race	Booster dose	Booster vaccine	COVID-19	Booster period
1	20-29	Female	White	3	BNT162b2	After	Sep - Dec 2021
2	40-49	Female	White	3	BNT162b2	No	Sep - Dec 2021
3	20-29	Female	Black	3	BNT162b2	No	Sep - Dec 2021
4	20-29	Female	White	3	BNT162b2	No	Sep - Dec 2021
5	40-49	Male	Asian	3	BNT162b2	No	Sep - Dec 2021
6	20-29	Female	Asian	3	BNT162b2	No	Sep - Dec 2021
7	20-29	Male	Asian	3	BNT162b2	After	Sep - Dec 2021
8	30-39	Female	White	3	BNT162b2	Before	Sep - Dec 2021
9	20-29	Male	White	3	BNT162b2	After	Sep - Dec 2021
10	20-29	Male	White	3	BNT162b2	Before	Sep - Dec 2021
11	30-39	Female	White	3	BNT162b2	No	Sep - Dec 2021
12	40-49	Female	White	3	BNT162b2	No	Sep - Dec 2021
13	20-29	Male	White	3	BNT162b2	After	Sep - Dec 2021
14	20-29	Female	White	3	mRNA1273	No	Sep - Dec 2021
15	20-29	Male	Black	3	BNT162b2	No	Sep - Dec 2021
16	20-29	Female	White	3	BNT162b2	No	Sep - Dec 2021
17	20-29	Male	Asian	3	BNT162b2	No	Sep - Dec 2021
18	20-29	Female	Asian	3	BNT162b2	Before	Sep - Dec 2021
19	30-39	Male	White	3	BNT162b2	No	Sep - Dec 2021
20	20-29	Male	White	3	mRNA1273	After	Sep - Dec 2021
21	20-29	Male	White	3	BNT162b2	After	Sep - Dec 2021
22	20-29	Female	White	3	mRNA1273	After	Sep - Dec 2021
23	20-29	Male	Asian	3	BNT162b2	No	Sep - Dec 2021
24	30-39	Female	White	3	BNT162b2	No	Sep - Dec 2021
25	30-39	Female	White	3	BNT162b2	No	Sep - Dec 2021
26	50-59	Male	White	3	BNT162b2	After	Sep - Dec 2021
27	60-69	Female	White	3	BNT162b2	After	Sep - Dec 2021
28	50-59	Female	White	3	BNT162b2	After	Sep - Dec 2021
29	40-49	Male	White	3	BNT162b2	After	Sep - Dec 2021
30	30-39	Female	White	3	BNT162b2	No	Sep - Dec 2021
31	50-59	Male	White	3	mRNA1273	Before	Sep - Dec 2021
32	20-29	Male	Asian	3	BNT162b2	No	Sep - Dec 2021
33	30-39	Male	White	3	mRNA1273	No	Sep - Dec 2021

34	20-29	Female	Mixed	3	mRNA1273	No	Sep - Dec 2021
35	50-59	Female	Black	3	mRNA1273	No	Sep - Dec 2021
36	70-79	Male	White	3	mRNA1273	No	Sep - Dec 2021
37	20-29	Male	White	3	BNT162b2	After	Sep - Dec 2021
38	30-39	Female	White	3	BNT162b2	No	Sep - Dec 2021
39	60-69	Female	White	3	BNT162b2	No	Sep - Dec 2021
40	20-29	Male	White	3	BNT162b2	Before	Sep - Dec 2021
41	30-39	Female	White	3	BNT162b2	Before	Sep - Dec 2021
42	30-39	Female	White	3	mRNA1273	After	Sep - Dec 2021
43	30-39	Female	White	3	mRNA1273	No	Sep - Dec 2021
44	50-59	Female	White	3	mRNA1273	No	Sep - Dec 2021
45	20-29	Female	White	3	BNT162b2	Before	Sep - Dec 2021
46	20-29	Female	White	3	mRNA1273	No	Sep - Dec 2021
47	20-29	Female	Asian	3	mRNA1273	No	Sep - Dec 2021
48	70-79	Male	Black	3	mRNA1273	No	Sep - Dec 2021
49	60-69	Female	Black	3	mRNA1273	Before	Sep - Dec 2021
50	60-69	Female	White	3	mRNA1273	No	Sep - Dec 2021
51	30-39	Male	White	3	mRNA1273	No	Sep - Dec 2021
52	40-49	Female	White	3	mRNA1273	After	Sep - Dec 2021
53	30-39	Female	White	3	mRNA1273	No	Jan - Apr 2022
54	30-39	Female	Black	3	mRNA1273	Before	Jan - Apr 2022
55	30-39	Male	White	3	BNT162b2	Before	Jan - Apr 2022
56	70-79	Male	White	4	mRNA1273	No	Jan - Apr 2022
57	50-59	Male	White	4	mRNA1273	No	Jan - Apr 2022
58	60-69	Female	Black	4	mRNA1273	No	Jan - Apr 2022
59	60-69	Female	Black	4	mRNA1273	After	Jan - Apr 2022
60	70-79	Female	White	4	mRNA1273	Before	May - Aug 2022
61	60-69	Male	White	4	mRNA1273	After	May - Aug 2022
62	60-69	Male	White	4	mRNA1273	No	May - Aug 2022
63	70-79	Male	White	4	mRNA1273	No	May - Aug 2022
64	60-69	Male	Black	4	mRNA1273	No	May - Aug 2022
65	80-89	Female	White	4	mRNA1273	Before	May - Aug 2022
66	60-69	Female	White	4	mRNA1273	No	May - Aug 2022
67	70-79	Female	White	4	mRNA1273	After	May - Aug 2022
68	60-69	Male	White	4	BNT162b2	Before	May - Aug 2022

Supplemental Figures

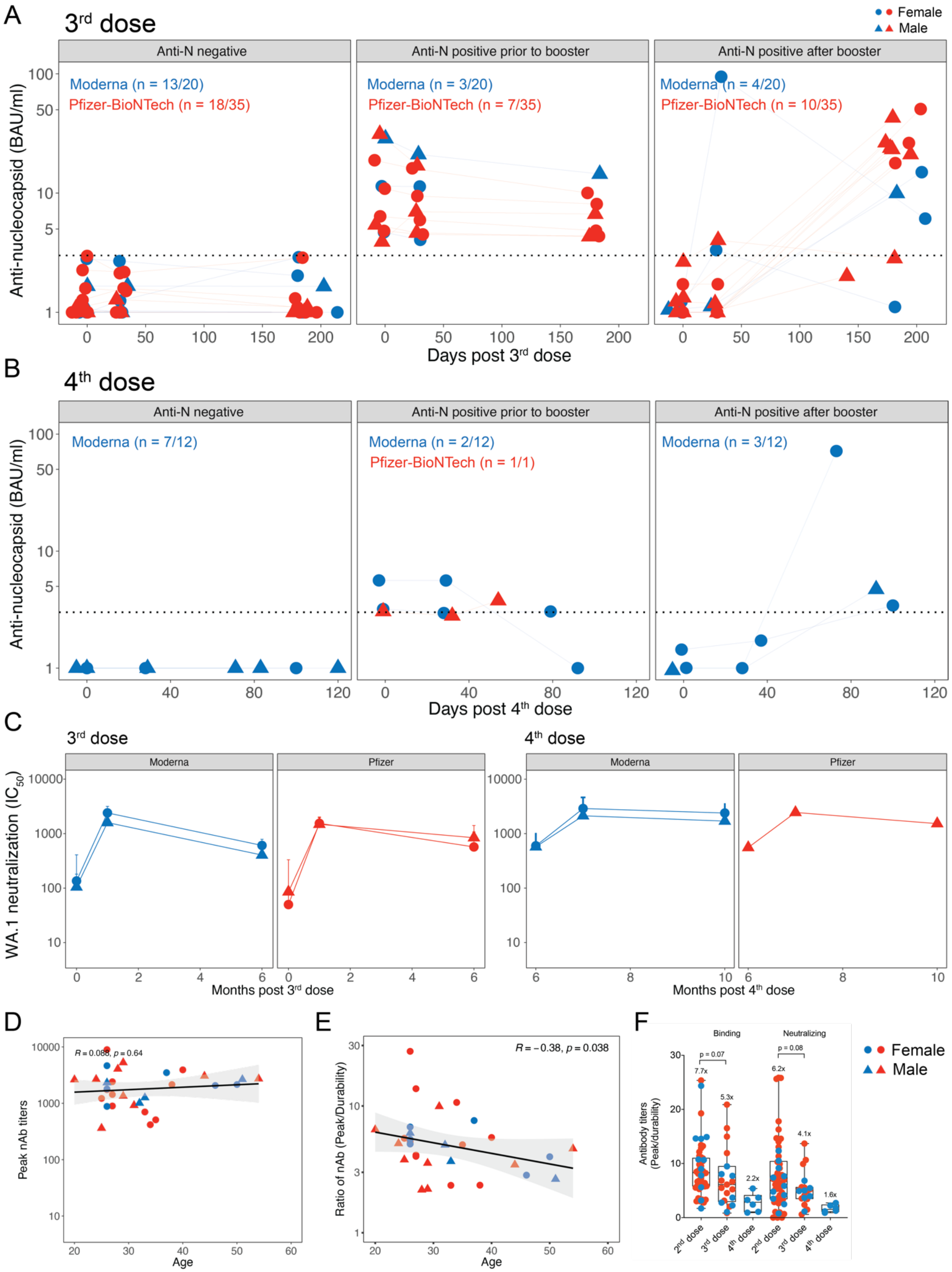

Supplemental Figure 1. Serum antibody responses following booster mRNA vaccinations. (A - B) Anti-N antibody response measured in the sera of participants who received 3 **(A)** or 4 **(B)** doses. The numbers within the plots show the proportion of individuals positive or negative for anti-N antibody response. **(C)** A summary of neutralizing antibody response against the ancestral strain in males versus females (Geomean + SEM). **(D - E)** Spearman's correlation between age and neutralizing antibody titers against the ancestral strain at peak **(D)** or ratio of peak versus durability **(E)**. **(F)** Fold change between binding and neutralizing antibody responses measured at peak (~1 month) versus durability (~6 months) after 2nd, 3^{rd,} or 4th doses. Each symbol represents a SARS-CoV-2 naïve participant. COVID-19^{+} individuals were removed from the analysis. The statistical significance was determined using the Mann-Whitney test. The data after 2nd vaccination were published previously by us.

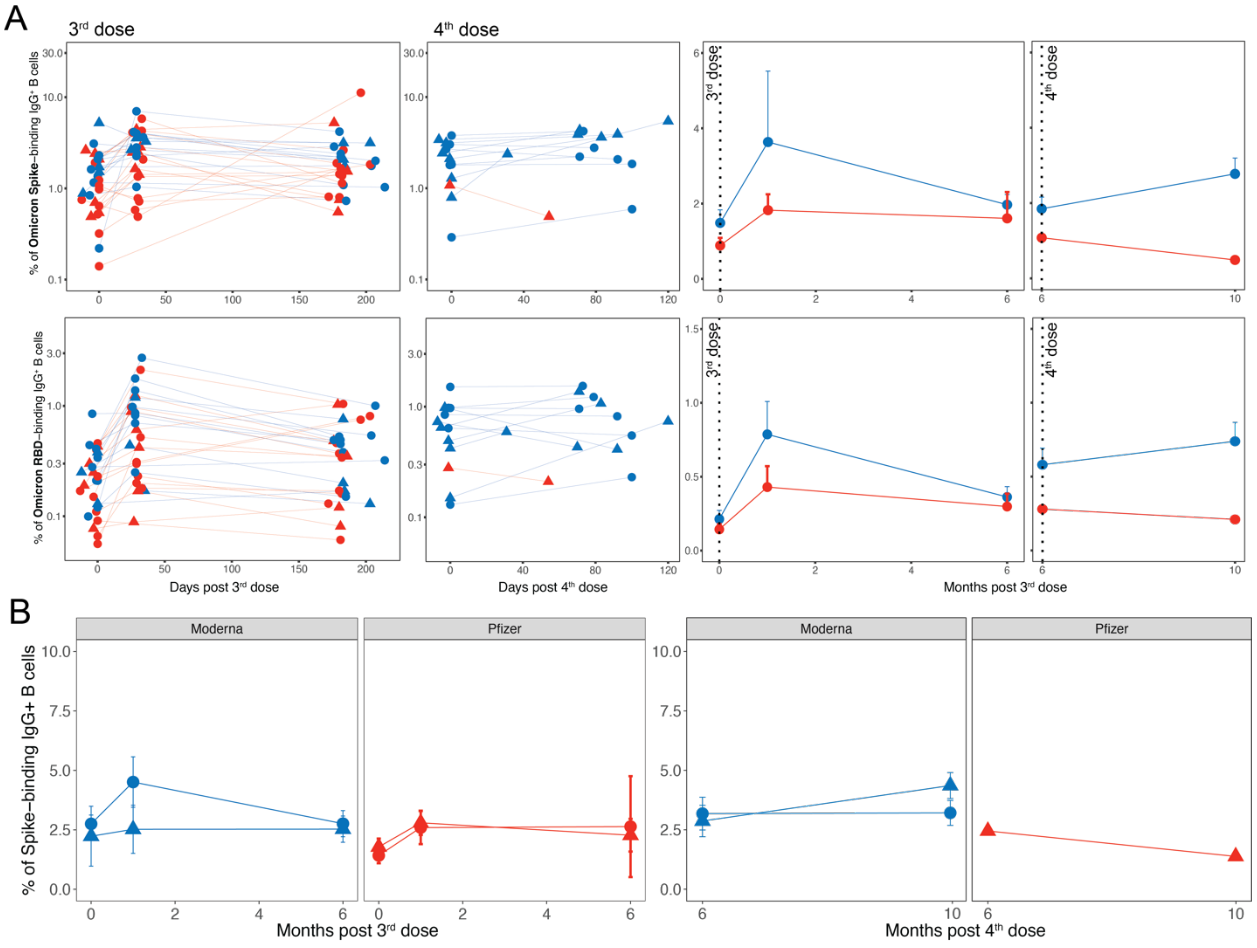

Supplemental Figure 2. Memory B cell response following booster mRNA vaccinations. (A) Frequency of Omicron BA.1 Spike (top panel) or RBD (bottom panel) specific memory B cells relative to $CD20^+$ IgD^- IgM^- B cells. Each symbol represents an individual (N = 28 after 3rd dose and 13 after the 4th dose). The statistical differences between time points were determined using the Wilcoxon matched-pairs signed rank test. The two graphs on the right show summary of the responses (Geomean + SEM). The statistical difference between the groups was determined using the Mann-Whitney test. (**B**) A summary of the frequency of Spike-binding memory B in males versus females. Data represent Geomean + SEM.

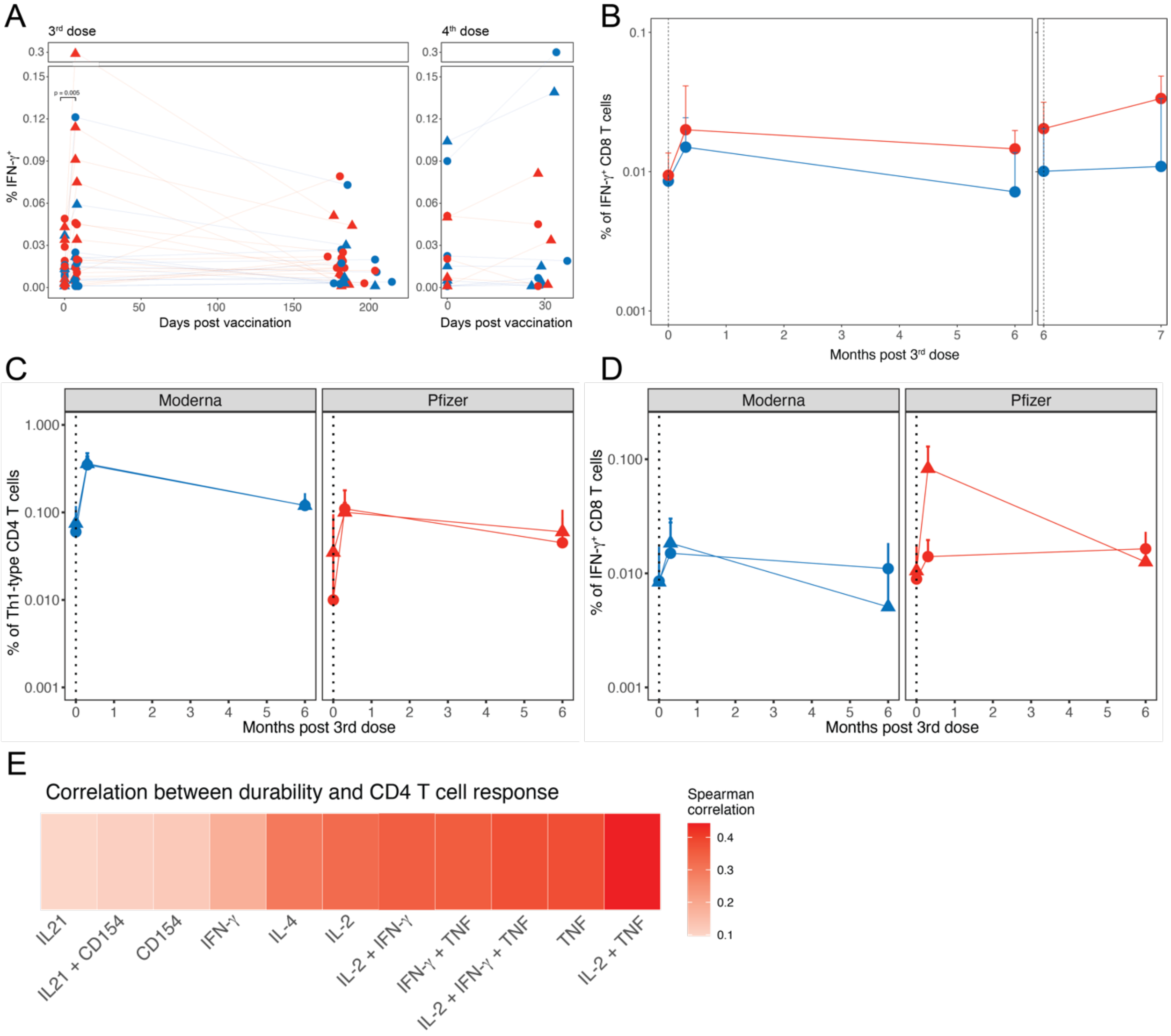

Supplemental Figure 3. T cell response following booster mRNA vaccinations. (A) Frequency of ancestral Spike-specific CD8 T cells producing IFN-γ. Each symbol represents an individual (N = 28 after 3rd dose and 13 after the 4th dose). **(B)** A summary of the frequency of Spike-specific CD8 T cells producing IFN-γ. Blue and red colors indicate Moderna and Pfizer-BioNTech vaccines. **(C – D)** A summary of the frequency of antigen-specific CD4 **(C)** and CD8 **(D)** T cells stratified by sex. Circles and triangles represent females and males, respectively. In all the summary plots, Geomean + SEM is shown. **(E)** Heatmap showing Spearman's correlation between ancestral Spike-specific CD4 T cell responses indicated on the horizontal axis and durability of the antibody response, defined using a ratio of neutralizing antibody response against the ancestral virus at the peak vs. durability time points. None of the correlations were statistically significant, i.e., $p_{adj} > 0.05$ after multiple corrections using the Benjamini-Hochberg method.
